# A reinforcement learning approach for selecting infill drilling locations considering long-term production planning in mining complexes with supply uncertainty

**DOI:** 10.1177/25726668241244930

**Published:** 2024-04-22

**Authors:** Zachary Levinson, Roussos Dimitrakopoulos

**Affiliations:** COSMO Stochastic Mine Planning Laboratory, Department of Mining and Materials Engineering, 5620 McGill University, FDA Building, 3450 University Street, Montreal, Quebec H3A 0E8, Canada

**Keywords:** infill drilling, simultaneous stochastic optimisation, reinforcement learning, stochastic programming, long-term planning, value of information

## Abstract

Simultaneous stochastic optimisation frameworks provide a method for optimising long-term production schedules in mining complexes that aim to maximise net present value and manage risk related to supply uncertainty. The uncertainty and local variability related to the quality and quantity of material in the mineral deposits are modelled with a set of stochastic orebody simulations, an input into the simultaneous stochastic optimisation framework. Infill drilling provides opportunities to collect additional information associated with the mineral deposits, which can inform future production scheduling decisions. A framework is developed for optimising infill drilling locations with a criterion that seeks areas that directly affect long-term planning decisions and requires the use of geostatistical simulations. Actor-critic reinforcement learning is applied to identify infill drilling locations in a copper mining complex using this criterion. The case study demonstrates that adapting production scheduling decisions given additional information has the potential to improve the associated production and financial forecasts and identifies a stable area for infill drilling.

## Introduction

A mining complex is an integrated supply chain designed to transform extracted materials from several mines into a set of valuable products for delivery to customers and the market (Dimitrakopoulos and Lamghari, 2022; Pimentel et al., 2010). Production scheduling decisions are optimised to maximise net present value (NPV) and, in cases where uncertainty is considered, minimise technical risk related to the uncertain material supply. Recent advancements in stochastic mathematical programming have demonstrated the benefits of simultaneously optimising long-term production scheduling decisions within a mining complex, while considering supply uncertainty (Goodfellow and Dimitrakopoulos, 2016, 2017; Montiel and Dimitrakopoulos, 2015, 2018). The production scheduling decisions are optimised simultaneously given the model of a mining complex and the various behaviour of the components within. Furthermore, the uncertainty and local variability of the material supply is accounted for and managed in the optimisation framework by using stochastic orebody simulations to represent the quantity and quality of materials in the ground (Goovaerts, 1997; Remy et al., 2009).

Infill drilling is important for capturing additional information about the mineral deposit that can potentially reduce sources of error by identifying boundaries and discrete ore zones to more accurately classify the mineral resources and reserves (Parker, 2012). Planning which areas to invest in infill drilling remains a major challenge in the mining industry because the outcomes of drilling are uncertain and the information collected may not necessarily cause a change in the long-term planning decisions. However, infill drilling in areas that provide valuable information for orebody modelling including geostatistical simulations can directly impact the outcome of the resulting long-term production schedule, potentially leading to improved production forecasts. To assess the influence of infill drilling on production scheduling decisions, a new framework is developed herein that quantifies improvements to the long-term production schedule based on infill drilling data. This provides an improved criterion for infill drillhole selection. Future infill drilling data is sampled from a stochastic realisation and used to update a set of stochastic orebody simulations (Jewbali and Dimitrakopoulos, 2011; Peattie and Dimitrakopoulos, 2013). The production schedule is then reoptimised considering new information to account for the forecasted improvements to the long-term production schedule.

Past work aiming to optimise infill drilling consider minimising estimation variance (Diehl and David, 1982; Gershon et al., 1988; Soltani and Safa, 2015), which is influenced by the geometry of the drillhole configuration, sample density, number of samples and covariance structure (Cressie, 1991). This ignores the critical impacts related to uncertainty and local grade variability of the attributes of interest (Delmelle and Goovaerts, 2009; Ravenscroft, 1992). Furthermore, additional drilling is often assumed to reduce grade uncertainty, however, Goria et al. (2001) and Dimitrakopoulos and Jewbali (2013) document cases where collecting additional information can lead to greater grade variability and uncertainty. This supports the suggestion to consider the potential impacts of infill drilling on the long-term mine plan.

Several approaches for optimising infill drilling decisions are founded upon geostatistical simulation techniques and have been introduced to overcome these limitations by accounting for the effects of different drilling patterns. Dirkx and Dimitrakopoulos (2018) apply a multi-armed bandit approach for selecting infill drilling patterns in a stockpile using a criterion that accounts for changes to material classification. Infill drilling patterns that cause the largest changes to the material classification of stockpiled material are selected as this is expected to reduce misclassification of these materials. To assess the value of infill drilling, additional data is retrieved from a stochastic realisation of future infill drilling data and used to re-simulate a set of stochastic orebody simulations. Boucher et al. (2005) use geostatistical simulations of correlated variables to observe the impact of drilling on the forecasted profits obtained while evaluating different stockpiling options. By virtually drilling a simulated realisation of the future infill drilling data (assumed to be the true deposit) and using the information to re-simulate the deposit with denser drilling patterns, an optimised configuration is determined by comparing block classifications and economic indicators. Kumral and Ozer (2013) propose an approach that aims to optimise the drilling configuration with a genetic algorithm that minimises the maximum interpolation variance by sampling the drilling information using future simulated drillhole data and available exploration data. Verly and Parker (2021) apply conditional simulations and confidence intervals to classify minerals as measured or indicated. This could be used as another criterion for infill drillhole planning. A limitation of these simulation-based methods is that they do not consider the relationship between the drilling information collected and potential effects on long-term mine planning and production scheduling, which may lead to additional value.

The approach presented herein introduces a new framework for optimising infill drilling decisions that consider long-term production scheduling decisions in the context of the simultaneous stochastic optimisation of mining complexes by evaluating potential improvements to the long-term production schedule and associated forecasts. Actor-critic reinforcement learning with deep neural networks (Lillicrap et al., 2015; Sutton and Barto, 2018) is applied to learn a policy for selecting infill drilling locations. The reinforcement learning policy guides the optimisation process to relevant drilling locations with a stochastic search, learning the areas that lead to the largest improvements in the production schedule through continuous trial-and-error. Stochastic orebody simulations are updated with ensemble Kalman filter (EnKF), which overcomes the requirement to re-simulate the entire deposit each time new additional information is retrieved (Evensen, 2003). Then, the long-term production schedule is simultaneously optimised considering the additional drilling information collected. Several recent works have demonstrated the successful performance of deep neural networks for a variety of optimisation problems (Kumar and Dimitrakopoulos, 2021; Kumar et al., 2020; Lillicrap et al., 2015; Mnih et al., 2015; Silver et al., 2017). Applying reinforcement learning for selecting infill drilling locations is advantageous as the stochastic search utilises contextual information from the mining complex to guide the optimisation process. Additionally, actor-critic methods work with continuous actions allowing the agent to select coordinates for drilling. The value of infill drilling in a mining complex is strongly related to the value of information, a frequent topic in decision theory (Bratvold et al., 2009; Yokota and Thompson, 2004). Froyland et al. (2018) discuss how additional infill drilling relates to these topics and highlights that the value gained from infill drilling can only be realised if it results in changes to production scheduling decisions. However, a method was not employed to address these challenges.

In the following sections, the proposed method based on actor-critic reinforcement learning and simultaneous stochastic optimisation is introduced for optimising infill drilling that provides potential to improve production and financial forecasting in mining complexes. Next, a case study at a copper mining complex demonstrates the key aspects of the proposed approach and the impact of selecting a set of infill drilling locations. Conclusions and recommendations for future work follow.

## Planning infill drilling with reinforcement learning and simultaneous stochastic optimisation

**Figure 1.**
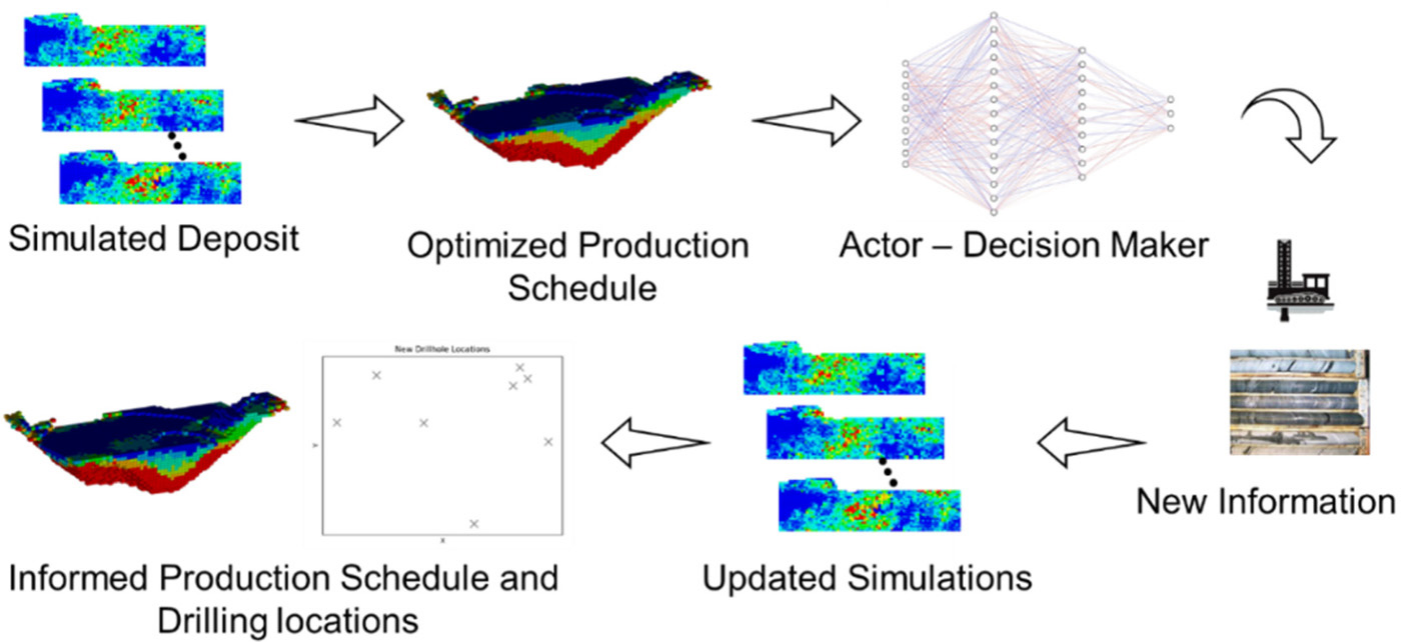
Primary workflow: (a) generate a set of equiprobable stochastic orebody simulations; (b) simultaneous stochastic optimisation of a mining complex; (c) sample drillhole from stochastic simulation (d) EnKF update; (e) retrieve drilling locations and adapted long-term production schedule.

The framework developed herein for planning infill drilling in mining complexes is outlined in this section. An overview of the workflow developed for infill drillhole selection is outlined in Figure 1.

The process begins by inputting a set of stochastic orebody simulations into the simultaneous stochastic optimisation framework. These stochastic orebody models represent the uncertain supply of material within the mineral deposit and are simulated using geostatistical techniques (Boucher and Dimitrakopoulos, 2009; Godoy, 2003). Then, the long-term production schedule is simultaneously optimised by jointly considering the extraction sequence, destination policy and processing stream decisions to maximise NPV and manage technical risk. The optimisation considers current information available for orebody modelling which accounts for the uncertainty and local variability of the material grades with a set of stochastic orebody simulations. The formulation for the simultaneous stochastic optimisation of mining complexes is detailed in Goodfellow and Dimitrakopoulos (2016, 2017). After obtaining the optimised production schedule given the information prior to infill drilling, a set of features is processed from the long-term production schedule forecasts and the stochastic orebody simulations to define the state of the mining complex. The state is observed by a reinforcement learning agent (or decision maker), which chooses an infill drilling location based on the current neural network policy denoted $\pi_{\theta }$ . The output action is a continuous output representing the coordinate of the drillhole collar (x, y) and whether to continue drilling. At the corresponding drillhole location, a sample is virtually drilled by retrieving drillhole data from a stochastic simulation of future infill drilling data that is considered here to represent the true deposit. EnKF is then applied to update the stochastic orebody simulations with the additional drilling information retrieved. After the update, the simultaneous stochastic optimisation approach is used to reoptimise the production schedule given new information, to determine if the update leads to any changes and improvements in the long-term production schedule and related forecasts. Lastly, the framework is tested several times using a different randomly selected simulation of future drillhole data to ensure similar locations are drilled independent of the realisation selected. Details related to each step are discussed in the subsequent sections.

### Reinforcement learning background

The reinforcement learning framework applied is an actor-critic reinforcement learning approach (Lillicrap et al., 2015; Sutton and Barto, 2018). The agent or decision maker interacts with a mining complex environment *E* over discrete timesteps to maximise the cumulative reward or return. The infill drilling approach is modelled as a Markov decision process consisting of a state space *S*, action space *A*, initial probability distribution with density $p_{1}(s_{1}),$ and stationary transition dynamics distribution with conditional density $p(s_{t+1}|s_{t},a_{t})$ and reward function *r*. Each timestep *t* the agent receives an observation of the state of the environment $s_{t}$ and selects an action $a_{t}$ . The environment responds by presenting new states $s_{t+1}$ to the agent along with a reward $r_{t}$ that is generated by the reward function. The agent behaviour is based on a learned policy $\pi_{\theta }$ that maps states to probabilities of selecting each possible action which can be accomplished by using a neural network policy with a parameter vector θ . The return $R_{t}$ that is obtained by the agent is the sum of discounted future rewards such that $R_{t}=\overset{T}{\underset{k=t}{\sum }}\;\gamma^{k-t}r(s_{k},\;a_{k})$ where γ∈(0,1] is the discount factor and *T* is the terminating timestep. The actor-critic reinforcement learning algorithm is used in this work to determine a policy that maximises the expected return from the starting state which is denoted by a performance function $J(\pi )=E[R_{1}|\pi ]$ .

Actor-critic reinforcement learning is applied largely due to its success with large continuous action spaces (Lillicrap et al., 2015), which works well for selecting infill drilling locations. In actor-critic reinforcement learning the policy is learned by adjusting the neural network parameters θ in the direction of the performance gradient $\nabla_{\theta }J(\pi_{\theta })$ (Sutton et al., 1999). This increases the probability of selecting actions that led to higher rewards and reduces the likelihood of selecting lower rewards. In actor-critic reinforcement learning, the value function is also learned. The value function is defined as the expected total discounted reward from a given state such that $V^{\pi }(s)=E[R_{t}|s_{t}=s;\pi ]$ and the action value function $Q^{\pi }(s,a)=E[R_{t}|s_{t}=s,\;a_{t}=a;\;\;\pi ]$ . In this case the value function represents the incremental improvement achieved by optimising the long-term production schedule given additional infill drilling data.

Additionally, when neural networks or other nonlinear function approximators are used to approximate a value function it can be unstable or potentially diverge. This is caused for two primary reasons including correlations in the sequence of the observations and the correlations between the action values and target values. These challenges are addressed using a replay buffer and a target network that are periodically updated (Lin, 1992; Mnih et al., 2013; Mnih et al., 2015).

### Defining the state space

The state of the mining complex environment that is used for selecting infill drilling locations is based on features derived directly from the optimised production schedule, orebody simulations and available information on site. These provide a numerical representation of the factors that were considered important in this work for drillhole selection. This information can include previously drilled locations, the scheduled period of extraction a drillhole will intersect and areas of higher or lower grade variability. Each feature is calculated based on the values obtained from the production schedule at timestep *t* and linear or non-linear transformation of the input parameters denote by the function ϕ:

(1) $$\begin{array}{c} s_{t}=\phi ({\mathrm{sched}}_{t}) \end{array}$$

where the ${\mathrm{sched}}_{t}$ is the production schedule of the mining complex given the information available at timestep *t*. These features are the input to the neural network policy $\pi_{\theta }$ that maps states to actions for infill drillhole selection. Through continuous trial and error, the most beneficial drilling locations are learned by optimising the parameters of the neural network using a stochastic gradient algorithm (Kingma and Ba, 2014). Unlike a stochastic search, context related to the mineral deposit and the mining complex production schedule can be incorporated into the optimisation process. This provides insight on which locations to drill within the mining complex based not only on the material uncertainty in the blocks themselves but on the distribution of metal generated downstream at different destinations during each production period. For example, years with higher material variability in terms of metal production may be targeted heavily depending on the impact on the production schedule.

### Updating simulations with ensemble Kalman filter (EnKF)

**Figure 2.**
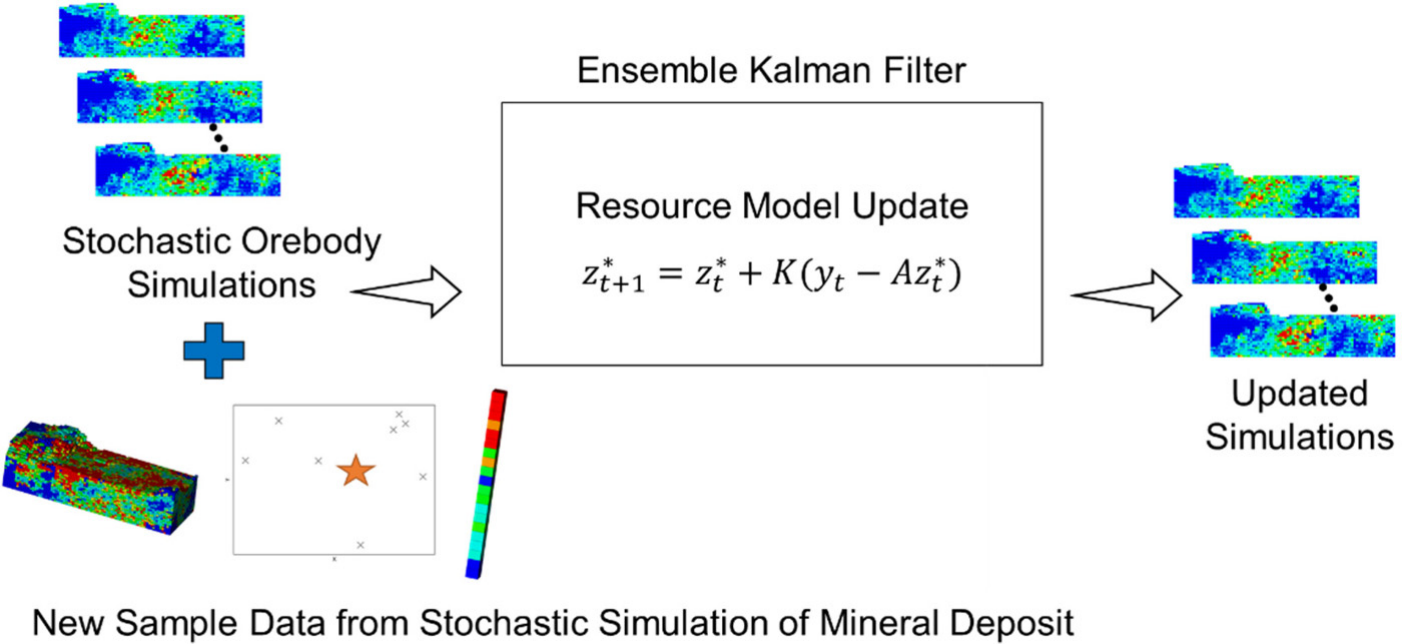
EnKF approach for updating stochastic simulations with additional drilling data.

An EnKF framework is applied where the stochastic orebody simulations are updated using new information collected from future infill drilling (Figure 2). The magnitude of the update is based on the error between the simulated realisations and observed drillhole data that is retrieved from a stochastic simulation of the drillhole data at the location selected. The sampled data represents the drilling data in a real deposit and different randomly selected simulations of the drillhole data are used over several runs to ensure that after each run of the optimisation that the drillholes selected are within similar areas. Benndorf (2020) describes the EnKF updating framework for mineral resource applications in further detail.

The EnKF framework provides an efficient process for updating a set of simulations S of a mineral deposit without re-simulating the entire deposit. A spatial random field of the attribute of interest is denoted Z(x) where each element $Z(x_{j})$ is a random variable associated with a block position at location $x_{j}$ for j={1,…,M} . Each realisation of the spatial random field and the corresponding material grade is denoted ***z*** *(*x***)***_t,ꝸ_*. This provides the predicted value at location $x_{j}$ given the information gathered up to timestep *t* and the realisation ꝸ, where t={1,…,T} and ꝸ ∈S . A timestep represents each time new information becomes available by selecting a new drillhole location. The spatial random field is limited to a neighbourhood of blocks in the deposit as it is only desirable to update parts of the realisation neighbouring the newly collected additional information. A *T* by *M* matrix A represents the contribution of each new drillhole sample up to the present timestep *t*. The rows of matrix A represent the contribution of each drillhole selected to the posterior simulated orebody realisations. When one of the *N* potential drilling locations are drilled all the locations intersected by drilling vertically contribute to the update and are included as a one in the matrix A . Each block location $x_{j}$ in the neighbourhood is then updated using the following update:

(2) $$z^{*}{\left(x\right)}_{t+1,ꝸ}=z^{*}{\left(x\right)}_{t,ꝸ}+\underset{\mathrm{Kalman}\;\mathrm{Gain}}{\underbrace{C_{t,t}A^{T}{\left(AC_{t,t}A^{T}+C_{v,v}\right)}^{-1}}}\underset{\mathrm{Innovation}}{\underbrace{\left(o_{t,ꝸ}-Az^{*}{\left(x\right)}_{t,ꝸ}\right)}}$$

where $z*(x{)}_{t+1,ꝸ}$ is the updated attribute for each realisation *ꝸ* after additional information is collected in timestep *t*. The covariance matrix $C_{t,t}$ is a NbyN matrix approximating the auto- and cross covariance of the random field between each element $Z(x_{j})$ . The Kalman gain matrix depicted in Equation 2 represents the weighting factor that determines the contribution of the update to those neighbouring locations considered in the update. This includes the covariance matrix $C_{v,v}$ of the measured error in the sample. The second component in the update is the innovation, which represents the error between the predicted outcome of the prior model and the observed drillhole value $o_{t,ꝸ}=o_{t}+\epsilon_{ꝸ}$ . The observation $o_{t,ꝸ}$ is the data value collected through drilling $o_{t}$ and a small noise term is added to consider measurement error. The EnKF approach uses the set of simulated scenarios to approximate the covariance matrix and increase computational efficiency. This updating framework is applied to the set of stochastic simulations after a drillhole is sampled.

### Actor-critic reinforcement learning

**Figure 3.**
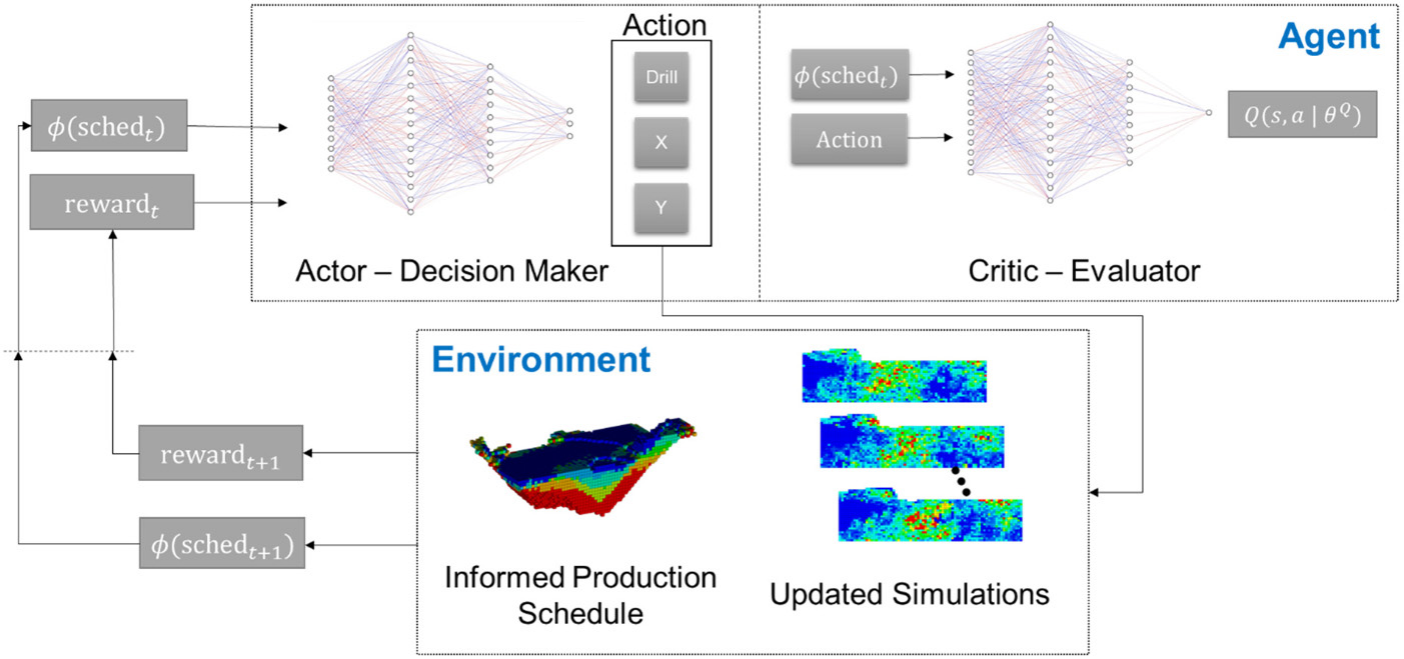
Actor-critic reinforcement learning framework for infill drillhole selection.

An off-policy actor-critic reinforcement learning agent is applied to optimise infill drilling. The actor-critic architecture is based on the policy gradient theorem (Peters et al., 2005; Sutton et al., 1999). During each timestep *t* the agent receives an observation from the mining complex $s_{t}=\phi ({\mathrm{sched}}_{t})$ , given the current long-term production schedule ( ${\mathrm{sched}}_{t})$ and a continuous action $a_{t}\in R^{3}$ is chosen to determine the collar coordinate (northing and easting) and whether drilling should be undertaken. The agent then receives a numerical reward $r_{t+1}$ from the mining complex based on the action taken by running the simultaneous stochastic optimisation framework given the new information. The learning framework is illustrated in Figure 3.

The actor-critic reinforcement learning algorithm uses an agent composed of two components. The actor learns a parametrised policy that maps the current state of the mining complex $s_{t}\;$ to an action $a_{t}=\mu (s_{t}|\theta^{\mu })$ and controls the decision-making process where $\theta^{\mu }$ denotes the parameters of the policy network. Similarly, the critic learns the action-value function $Q(s_{t},a_{t}|\theta^{Q})$ through a second set of parameters $\theta^{Q}$ . The action value-function is used to approximate the expected value of taking an action $a_{t}$ in state $s_{t}$ parametrised by $\theta^{Q}$ . Upon initialisation, the actor $\mu (s_{t}|\theta^{\mu })$ and critic network $Q(s_{t},a_{t}|\theta^{Q})$ are randomly initialised with weights $\theta^{\mu }$ and $\theta^{Q}$ , respectively. A duplicate of each of these networks are created as a target network to stabilise learning denoted $\mu^{\prime }$ and $Q^{\prime }$ with initial weights $\theta^{\mu^{\prime }}$ and $\theta^{Q^{\prime }}$ , respectively. Lastly, a replay buffer denoted R is initialised. The replay buffer is used to train the network off-policy (Lin, 1992). As the agent interacts with the mining complex, each experience it obtains is stored as a tuple ( $s_{t},a_{t},r_{t},s_{t+1})$ in a buffer that contains information from the most recent interaction with the mining complex. These experiences or transitions are randomly sampled to minimise the correlations between samples and update the neural network parameters.

Each episode of learning is a sequence of states, actions and rewards that end in a terminal state. In the mining complex, the actor decides which locations to infill drill based on the input features and proceeds until the terminal state is reached. The terminal state can occur when the agent decides to no longer continue drilling or a budgetary constraint is exceeded. A random process N introduces noise to each action to provide adequate exploration of the solution space. This is defined by the exploratory policy $\mu^{\prime }(s_{t})=\mu (s_{t}|\theta^{\mu })+N$ . In addition, the initial state of the environment is received by calculating a set of features $s_{1}=\phi ({\mathrm{sched}}_{1})$ derived from the optimised production schedule ${\mathrm{sched}}_{1}$ that considers the information available prior to additional drilling. The algorithm proceeds as follows:

1. An action $a_{t}=\mu (s_{t}|\theta^{\mu })+N_{t}$ is selected based on the current policy network and exploration noise.
2. The agent selects the nearest drillhole location to the output coordinates given by the agent's action $a_{t}$ . If drilling occurs, the drillhole is sampled and used to update a set of simulated realisations $S_{t}$ using EnKF. This results in a new set of simulated realisations $S_{t+1}$ updated to account for the information collected through infill drilling. If the agent decides not to drill step three of the process is skipped and a reward of 0 is received as drilling is not completed.
3. After drilling, a reward $r_{t}$ is generated by simultaneously optimising the mining complex given new information. The objective function used for optimising the long-term production schedule maximises NPV and minimises deviations from production targets by applying a set of penalties (PEN), see Appendix A. Considering this objective, the reward is computed by determining the difference in the objective function between the optimised long-term production schedule given new information ( ${\mathrm{sched}}_{t+1}$ ) and the schedule obtained prior to collecting new information ( ${\mathrm{sched}}_{t}$ ) minus the cost of drilling (DC) :
(3) $$\begin{array}{c} r_{t}=E_{S_{t+1}}[\mathrm{NPV}-\mathrm{PEN}|\mathrm{sche}d_{t+1}]-E_{S_{t+1}}[\mathrm{NPV}-\mathrm{PEN}|\mathrm{sche}d_{t}]-\mathrm{DC} \end{array}$$

Note that equation (3) accounts for the most up-to-date information available ( $S_{t+1})$ when evaluating the reward. The state $s_{t+1}$ can then be reconstructed using the optimised schedule that considers new information and the updated stochastic simulations. The optimised schedule given new information will likely change due to the difference in input and a positive reward is realised if the improvement in the objective function overcomes the cost of additional drilling.

4. Each transition in the environment is stored in a replay buffer R as a tuple ( $s_{t},a_{t},r_{t},s_{t+1})$ and a batch of *P* transitions are sampled ( $s_{i},a_{i},r_{i},s_{i+1})∼R$ to facilitate learning. The return is calculated using the target network that combines the reward received in timestep *i* and the predicted action-value provided by the critic target network.
(4) $$\begin{array}{c} y_{i}=r_{i}+Q^{\prime }(s_{i+1},\mu^{\prime }(s_{i+1}|\theta^{\mu^{\prime }})|\theta^{Q^{\prime }}) \end{array}$$
5. The critic and policy networks are updated by minimising the loss *L* and using the sampled policy gradient $\nabla_{\theta^{\mu }}J\;$ to improve the policy, respectively.
(5) $$\begin{array}{c} L=\frac{1}{P}\underset{i}{\sum }\;{\left(y_{i}-Q(s_{i},a_{i}|\theta^{Q})\right)}^{2} \end{array}$$
(6) $$\begin{array}{c} \nabla_{\theta^{\mu }}J\;\approx \frac{1}{P}\underset{i}{\sum }\;\nabla_{a}Q(s,a|\theta^{Q}){|}_{s=s_{i},a=\mu (s_{i})}\nabla_{\theta^{\mu }}\mu (s|\theta^{\mu }){|}_{s=s_{i}} \end{array}$$
6. Lastly, the target networks are then updated using the updated policy and critic network parameters:
(7) $$\begin{array}{c} \theta^{Q^{\prime }}\leftarrow \tau \theta^{Q}+(1-\tau )\theta^{Q^{\prime }} \end{array}$$
(8) $$\begin{array}{c} \theta^{\mu^{\prime }}\leftarrow \tau \theta^{\mu }+(1-\tau )\theta^{\mu^{\prime }} \end{array}$$
  where τ∈(0,1] is the weighting factor that gradually updates each target network to be closer to current network parameters.

The process is repeated until the agent converges or the maximum number of trials are reached. PyTorch is used for training the neural network (Paszke et al., 2019). A flowchart for the method can be found in Appendix B.

### Testing the agent

After learning is completed, the trained actor (neural network policy) is used to select additional infill drilling locations. The stochastic orebody simulations of the deposit prior to additional information are provided as input along with the stochastically optimised production schedule prior to additional information. The random process noise used for action exploration is eliminated and drillholes are selected until the budget constraint is reached or the trained actor decides it is no longer valuable to continue drilling. The resulting output is the infill drilling configuration that leads to the largest improvement in NPV and/or reduction of technical risk based on the current policy. The learning process is repeated several times with different stochastic simulations of the mineral deposit for sampling drillholes to ensure stability of the results and that similar locations are found independent of the stochastic realisation that is sampled.

## Case study in a copper mining complex

The framework developed for selecting additional infill drilling locations is tested in an operating copper mining complex with an open pit mine, several processing streams, stockpiles and a waste dump facility. The objective of the proposed framework is to determine a set of infill drilling locations that maximise NPV and minimise the risk of deviating from production targets given a fixed drilling budget.

**Figure 4.**
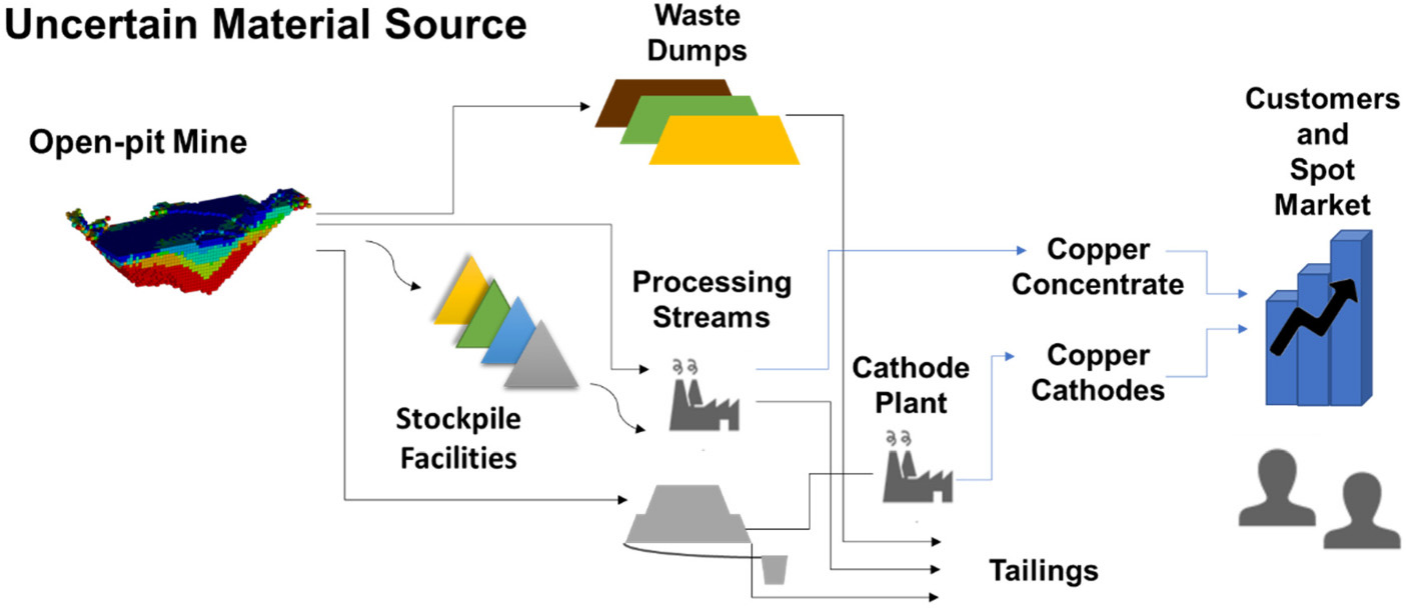
The copper mining complex.

The mining complex considered is shown in Figure 4, which includes the allowable material flows from the open pit mine to the customers and the market. A set of stochastic orebody simulations are used to represent the uncertain material supply. The simulated attributes considered include total copper and soluble copper. There are two processing streams with different recoveries. The process plant produces copper concentrate and can recover sulphide and oxide materials. The heap leach destination is used to recover copper from oxide materials and produces a copper cathode product after treatment at a cathode plant. In addition, a stockpile facility is included in the mining complex to allow material to be stored and processed in later periods. The extraction sequence, destination policy and process stream decisions are optimised with the simultaneous stochastic optimisation framework and the resulting production schedule is the initial input to the infill drilling optimisation framework. Lastly, the economic parameters for optimising the mining complex are shown in Table 1 (scaled for confidentiality reasons).

**Table 1.** Economic parameters.

Parameter (unit)	Value
Selling price ($/t)	2998
Ore processing ($/t)	8
Leach processing cost ($/t)	3.2
Mining cost ($/t)	1
Stockpile rehandle cost ($/t)	0.2
Discount rate (%)	10

The case study considers a set of 10 stochastically simulated orebody realisations with a block size of 20 × 20 × 15 $m^{3}$ as input and 108,000 blocks (Boucher and Dimitrakopoulos, 2009; Godoy, 2003; Goovaerts, 1997). Uncertainty in the boundaries of the geological domains is not considered in this case study, only the grades are simulated. Future case studies could also consider simulating the boundaries of geological domains to account for other uncertain mineral deposit characteristics that may also influence drilling, similar to how they can influence production planning in mining complexes (Morales, 2021; Morales and Dimitrakopoulos, 2024). To account for the uncertain mineral deposit, several different geostatistical simulations of future infill drilling data are considered to represent potential samples retrieved from a real mineral deposit. These simulations are different than the stochastic orebody simulations used for optimising the mining complex. Lastly, there is a budgetary constraint of $1 M and using this budget the algorithm can select from 3640 potential drilling locations.

**Figure 5.**
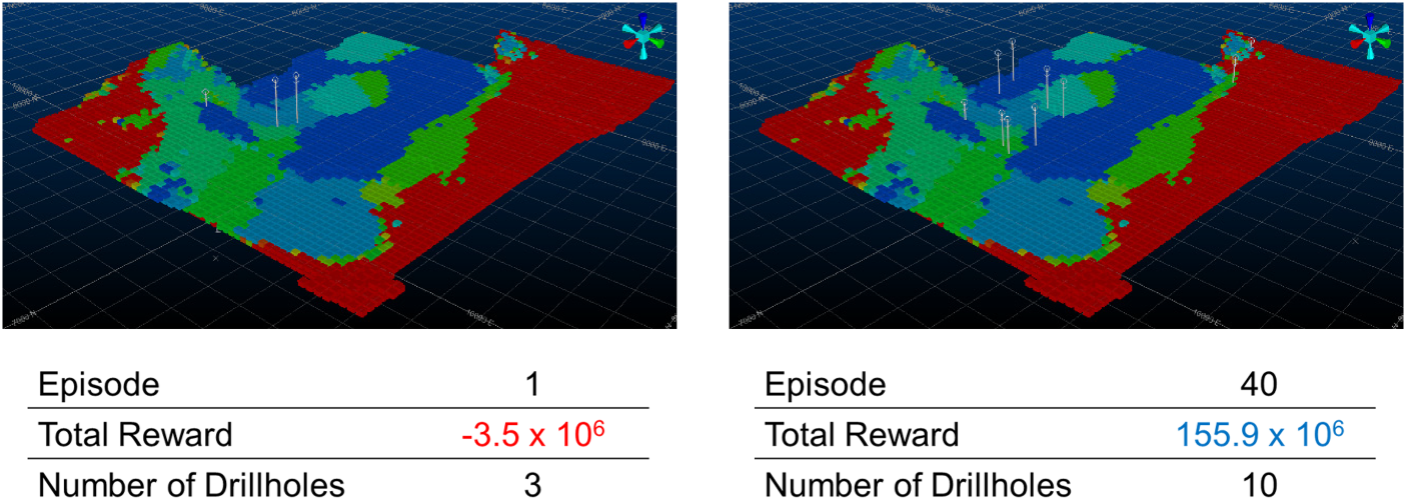
Training examples for different episodes including the number of drillholes drilled and the total cumulative reward obtained.

During training, the actor-critic reinforcement learning agent is left to interact with its environment to learn a set of infill drilling locations that provide the largest improvement in the long-term production schedule. Two example episodes of training the reinforcement learning approach are illustrated in Figure 5. The image shows the initial production schedule generated given the information prior to additional infill drilling and the periods of extraction are indicated by the colours of each block. Marked using a circular survey collar location are the different drilling locations determined by the actor during each episode of training. The total reward quantifies the improvement in the objective function obtained through collecting additional information. Additionally, the number of holes drilled in each episode are listed. The number of drillholes selected changes between each episode as the reinforcement learning agent also decides when to stop drilling. The cumulative reward obtained varies significantly depending on where drilling is commenced and the potential value added by adapting the production schedule to new information. Finally, the trained actor is used to select the number of drillholes and appropriate locations. The stochastic orebody simulations are updated using EnKF and a new production schedule is created using the simultaneous stochastic optimisation framework.

**Figure 6.**
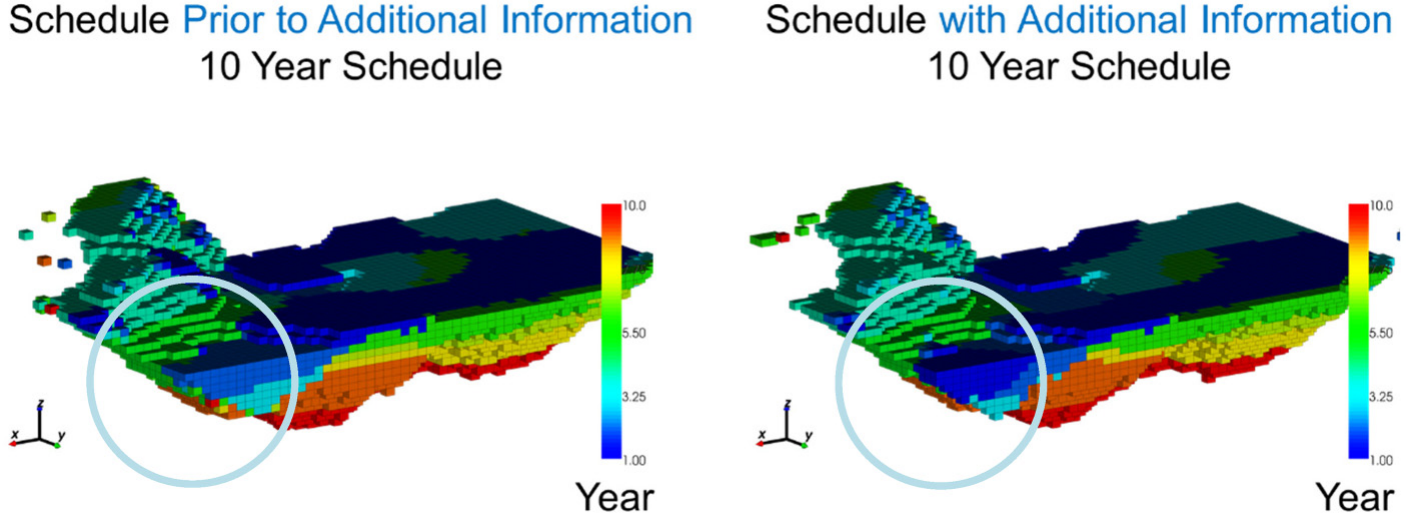
Production schedule: (left) prior to additional information; (right) after additional drilling.

**Figure 7.**
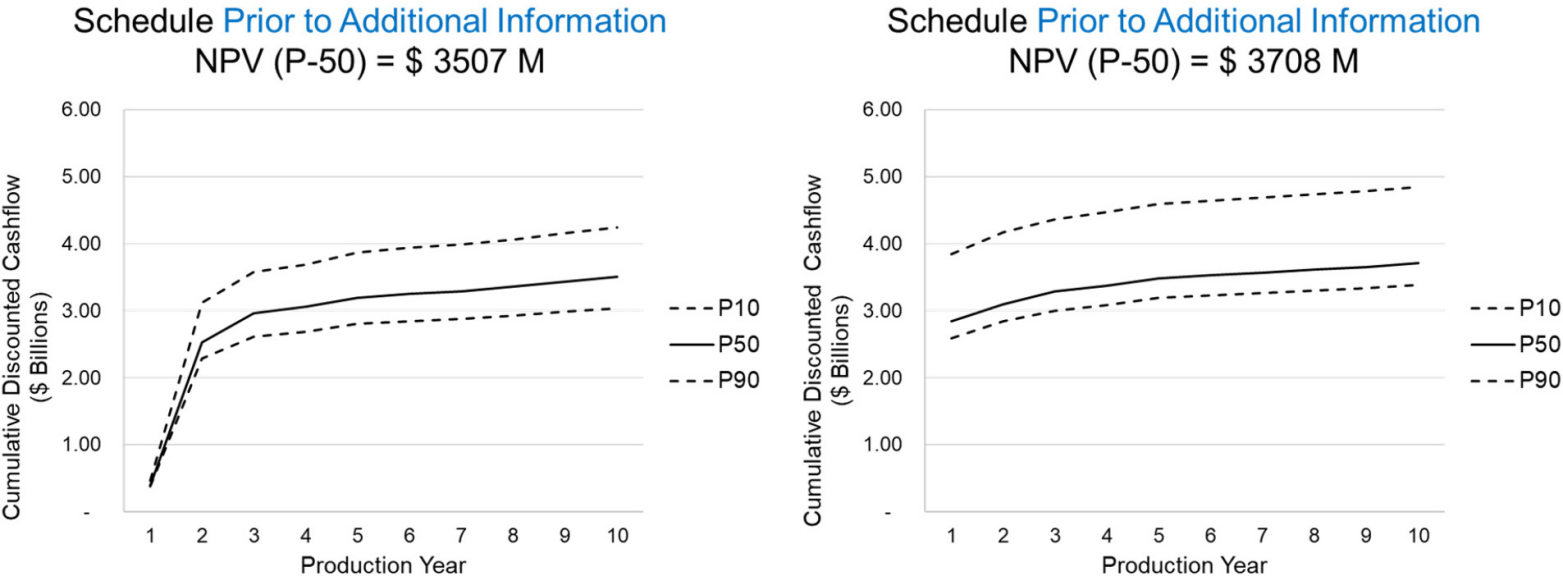
Cumulative discounted cashflows given the schedule: (left) prior to additional information; (right) after additional drilling.

**Figure 8.**
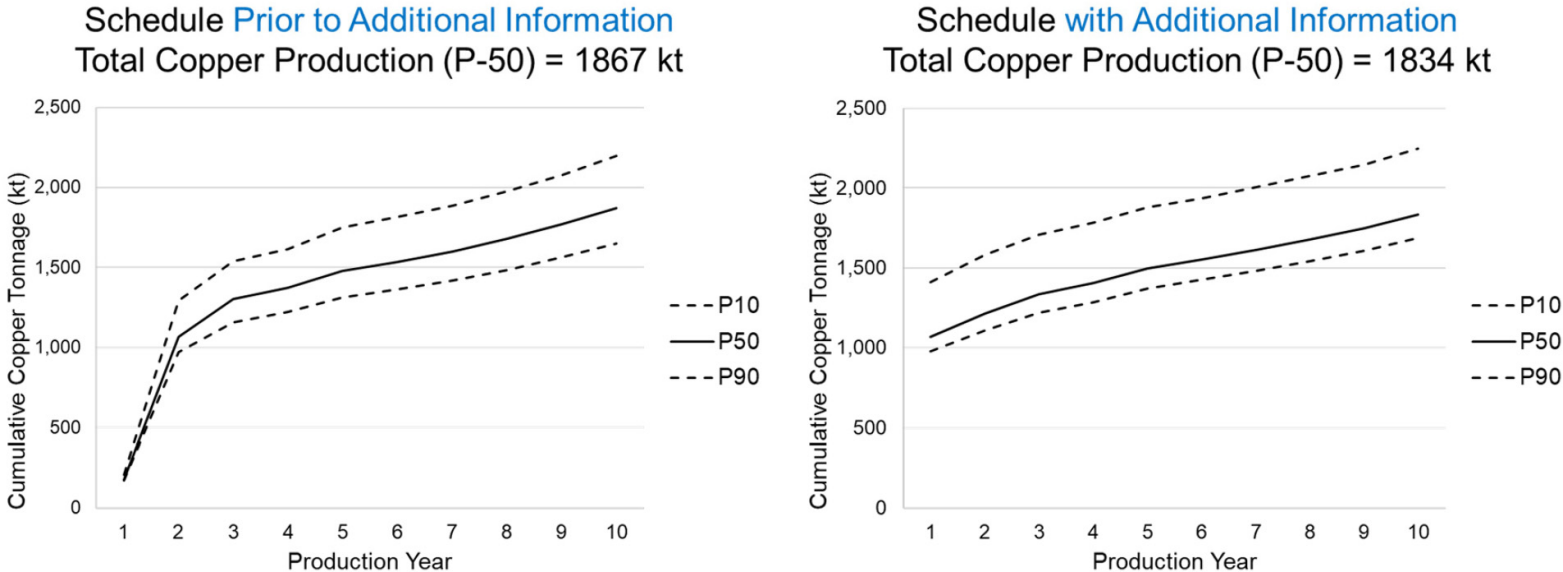
Total copper production given the schedule: (left) prior to additional information; (right) after additional drilling.

In Figure 6, the resulting production schedule prior to new information (left) is compared to the production schedule with additional information (right). The primary findings reveal that the extents of the final pit are similar between the two production schedules, however, the extraction sequence over the 10-year schedule changes significantly. In the main areas of drilling, marked with a circle, the early extraction sequence decisions change due to additional drilling information by adapting to an area of lower risk to generate further value for the mining complex. As a result of extracting this area earlier in the mine life other areas are pushed back to later periods. This is noticeable in the central part of the deposit as the simultaneous stochastic optimisation approach adapts the schedule to manage production targets and increase NPV. In Figure 7, the schedule that adapts to additional infill drilling information, which results in a 5.7% increase in the forecasted NPV. Furthermore, the additional information changes the resulting production forecasts leading to a 33 kt decrease in recovered copper at the process plant over 10 production years which is shown in Figure 8. This leads to a significant improvement to the long-term production schedule forecasts which is only obtained by adapting the production schedule to potential new information that is collected via infill drilling. Mining higher grade materials earlier with a higher confidence during the first two production years helps improve the overall production cashflows as drilling provides further confidence for mining these areas taking advantage of the time value of money.

**Figure 9.**
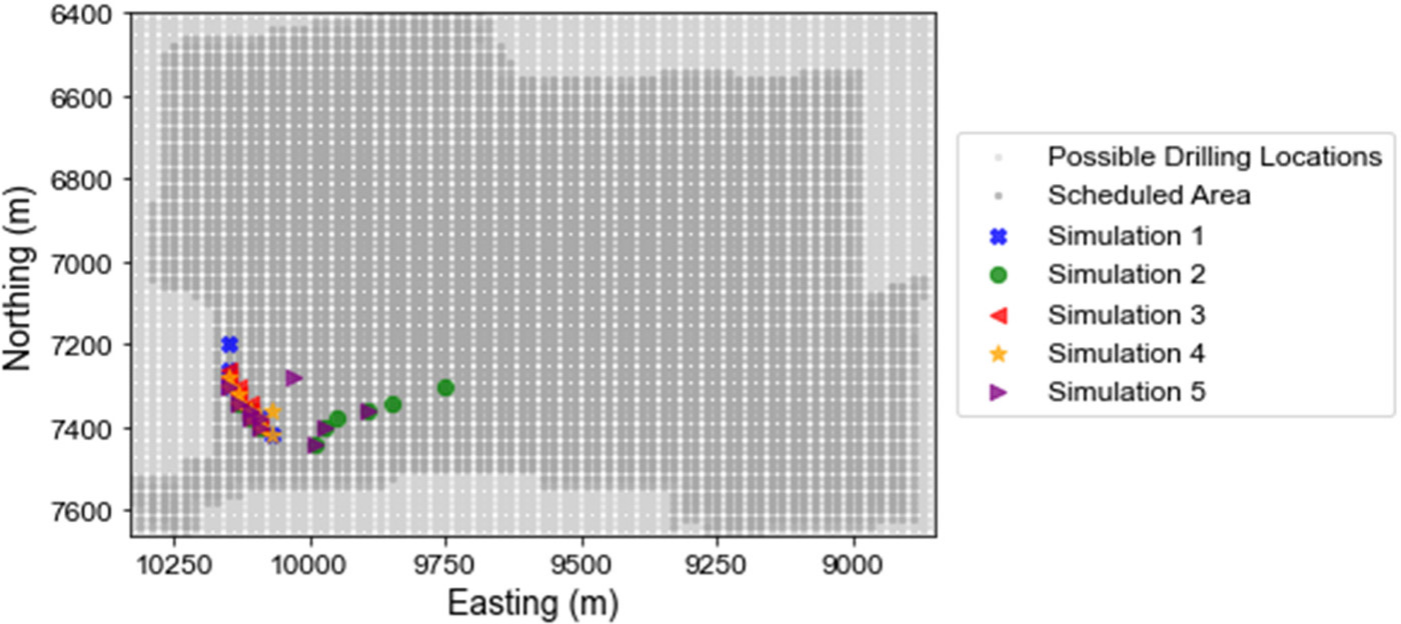
The infill drilling locations selected are shown for each run of the algorithm that use a different stochastic simulation to represent the true drillhole data. The light grey shows the possible drilling locations and the dark grey shows the scheduled areas.

Five different randomly sampled geostatistical simulations of future infill drilling data are used to represent the potential mineral deposit in five different runs. The proposed framework is run five times where the infill drilling data is retrieved from the respective simulation. The repeated testing of the proposed process using different simulations to represent the mineral deposit shows that similar areas are drilled and identifies a stable area for infill drilling (Figure 9). The drillholes selected in each run are shown in Figure 9, light grey points show all possible drilling locations in the mining complex and the dark grey region shows the extent of the scheduled areas in the long-term production schedule. The drillholes are highly localised in the northeast portion of the open pit mine and there is considerable overlap in the drillhole locations when using different simulations to represent the sampled infill drilling data. Furthermore, the drilling area is contained within a 250 m × 500 m area. Therefore, the drillhole selection framework identifies a drilling area in a stable way that is not affected by using different or additional realisations.

## Conclusions

A new approach has been proposed to determine infill drilling locations in mining complexes that uses actor-critic reinforcement learning. Reinforcement learning guides the framework to valuable drilling locations with a new criterion that links infill drilling to its impact on long-term mine production scheduling decisions. Simultaneous stochastic optimisation is applied to quantify the expected improvements to the long-term production schedule based on the new information collected. This provides insight on where drilling should occur to understand the complexity of the mineral deposits by updating the stochastic orebody simulations that are used as inputs for long-term production planning. The proposed approach was tested in a copper mining complex where the results demonstrate significant improvements to the forecasted NPV of the project by adapting the schedule to potential future infill drilling data. This method eliminates the need to use conversion indicators and other proxies to the production schedule value and directly considers the influence of drilling on production scheduling decisions. Actor-critic reinforcement learning is applied to optimise the infill drilling locations and takes advantage of key criteria from the mining complex to select infill drilling locations including considerations related to the uncertainty and local variability of the mineral deposits. Future work should consider allowing the proposed approach to select drillholes with different azimuth and dips to increase the flexibility in the drilling direction. In addition, supplementary information related to the deposit and production schedule could be explored to provide additional context for the drillhole selection process and improve the reinforcement learning agent's ability to distinguish valuable locations to drill.

## Figures and Tables

**Figure 10.**
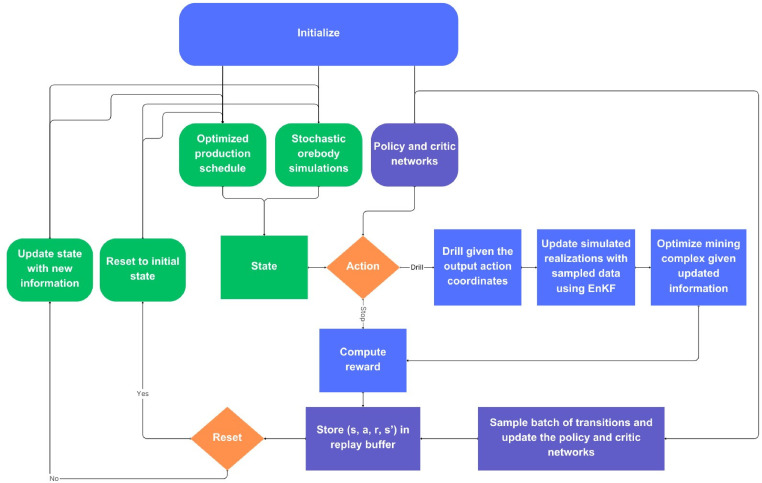
Flowchart of infill drilling approach with actor-critic reinforcement learning.

## Appendix A: Simultaneous stochastic optimisation.

The simultaneous stochastic optimisation framework outlined in Goodfellow and Dimitrakopoulos (2016) uses the following objective function:

(A.1)
$$\begin{array}{c} \max\underset{\mathrm{Discounted}\;\mathrm{Revenues}\;\mathrm{and}\;\mathrm{costs}}{\underbrace{\frac{1}{\left|S\right|}\underset{i\in S\cup P\cup M}{\sum }\;\underset{t\in T}{\sum }\;\underset{h\in H}{\sum }\;\underset{s\in S}{\sum }\;p_{h,i,t}v_{h,i,t,s}}}\\ -\underset{\mathrm{Risk}\;\mathrm{discounted}\;\mathrm{penaties}\;\mathrm{for}\;\mathrm{deviations}}{\underbrace{\frac{1}{\left|S\right|}\underset{i\in S\cup P\cup M}{\sum }\;\underset{t\in T}{\sum }\;\underset{h\in H}{\sum }\;\underset{s\in S}{\sum }\;(c_{h,i,t}^{+}d_{h,i,t,s}^{+}+c_{h,i,t}^{-}d_{h,i,t,s}^{-})}} \end{array}$$

The optimisation objective maximises the profit accounting for costs and revenues that are incurred to generate the products in the first term and minimises the penalties from deviations from production targets using the second term. Further details regarding the notation are shown in Table 2.

**Table 2.** Notation and definitions for objective function.

Notation	Definitions
S	A set of jointly simulated scenarios where a simulation is denoted by *s* and defines a realisation or sampling from all sources of uncertainty.
M	A set of mines, indexed by *i*.
H	A set of hereditary attributes, indexed by *h*.
T	A set of production time periods usually years, indexed by *t*.
S	A set of stockpiles, indexed by *i*.
P	A set of processing facilities, indexed by *i*.
$c_{h,i,t}^{+/-}$	Penalty costs for ± deviations from targets for each hereditary attribute h∈H at location i∈S∪P∪M in period t∈T.
$d_{h,i,t,s}^{\pm }$	± deviations from targets for each hereditary attribute h∈H at location i∈S∪P∪M in period t∈T and scenario s∈S .
$v_{h,i,t,s}$	The quantity of a hereditary attribute h∈H at location i∈S∪P∪M in period t∈T and scenario s∈S .
$p_{h,i,t}$	The quantity of a hereditary attribute h∈H at location i∈S∪P∪M in period t∈T.
